# Characterization of the Mitochondrial Localization of the Nuclear Receptor SHP and Regulation of Its Subcellular Distribution by Interaction with Bcl2 and HNF4α

**DOI:** 10.1371/journal.pone.0068491

**Published:** 2013-07-09

**Authors:** Yuxia Zhang, Li Wang

**Affiliations:** Departments of Medicine and Oncological Sciences, Huntsman Cancer Institute, University of Utah School of Medicine, Salt Lake City, Utah, United States of America; Univeristy of California Riverside, United States of America

## Abstract

The nuclear receptor small heterodimer partner SHP was shown recently to translocate to the mitochondria, interact with Bcl2, and induce apoptosis in liver cancer cells. However, the exact mitochondrial localization of SHP remains to be determined. In addition, the detailed interaction domains between SHP and Bcl2 have not been characterized. Using biochemistry and molecular biology approaches, we demonstrate that SHP is localized to the mitochondrial outer membrane. Interestingly, compared with the full-length SHP, the N-terminal deleted protein exhibits increased expression in the mitochondria and decreased expression in the nucleus. GST pull-down assays demonstrate that the interaction domain of SHP shows the strongest interaction with Bcl2. Furthermore, the interaction of Bcl2 with SHP is completely abolished by deletion of the Bcl2 transmembrane domain (TM), whereas deletion of the Bcl2 BH1 domain enhances the interaction. As expected, AHPN, a synthetic SHP ligand, markedly augments the direct protein-protein interaction between Bcl2 and SHP. Ectopic expression of hepatocyte nuclear factor 4 alpha (HNF4α) results in exclusive nuclear translocation of SHP proteins that contain either the full-length or the N-terminal domain, but has a minimal effect on the subcellular distribution of SHP protein containing only the interaction domain or repression domain. These results indicate that the N-terminal domain of SHP is important for itsnuclear translocation via HNF4α. Overall, this study provides novel insights into the domains of SHP that are critical for its shutting between different subcellular compartments.

## Introduction

The nuclear receptor (NR) superfamily consists of 48 members in humans and 49 in mice [1], [2]. Small heterodimer partner (*SHP*, NR0B2) is an orphan nuclear receptor that is unique in lacking the DNA binding domain commonly seen in other NRs [3]. Structurally, SHP contains an N-terminal domain (N), an interaction domain (Int) and a repression domain (Rep) [4]. Functionally, SHP is pleiotropic receptor that plays critical roles in several metabolic diseases [5], including disorders of bile acid homeostasis [6]–[8], lipid metabolism in adipose tissue [9], [10] and liver [11]–[17], and Toll-like receptor (TLR) signaling [18]. The action of SHP is through its repression of a complex set of genes in multiple pathways [3]. Recent studies provide strong evidence for a tumor suppressor function of SHP in liver cancer. Specifically, deletion of the Shp gene promoted spontaneous hepatoma formation in mice [19], and overexpression of Shp inhibited hepatocyte proliferation and activated hepatocyte apoptosis [20]. SHP expression was also markedly diminished in human HCC specimens due to promoter hypermethylation [21]. The newly identified role of SHP in the regulation of DNA methylation [22], [23] raised the intriguing possibility of using SHP as a potential therapeutic target.

Apoptosis is essential for maintenance of tissue homeostasis and depends on the activation of caspases, which is tightly regulated by the B-cell lymphoma protein 2 (Bcl2) family of proteins [24]. The Bcl2 family contains three functional subgroups, namely the sensors, guardians, and effectors [25]. Bcl2, the founding member of the family, is localized to the membrane surface of the mitochondria, ER, and nucleus [26]. Bcl2 is an antiapoptotic family member containing four conserved α-helical motifs known as Bcl2 homology (BH1-4) domains, as well as a transmembrane domain (TM) [27]. We recently discovered a cytoplasmic function of SHP in the induction of apoptosis via its interaction with Bcl2 in mitochondria to induce cytochrome *c* release [20]. However, the specific mitochondrial location of SHP remains elusive. In addition, the detailed interaction domains between SHP and Bcl2 have not been characterized.

The nuclear receptor hepatocyte nuclear factor 4 alpha (HNF4α) is a key regulator of pathways associated with various metabolic diseases [28]. The AF2 surface of HNF4α in the C-terminal region was reported to interact with SHP based on mammalian two-hybrid mapping [4]. Nonetheless, the domain that is critical for SHP nuclear translocation via its interaction with HNF4α remains to be identified.

Using biochemical and molecular biological approaches, we demonstrate in the present study that SHP is localized to the mitochondrial outer membrane. We further show that the interaction domain of SHP and the TM domain of Bcl2 are critical for their protein-protein interaction. Lastly, we show that the N-terminal domain of SHP is important for its nuclear translocation via interaction with HNF4α. Dissecting out the functional domains responsible for subcellular localization and expression of SHP provides essential information on the molecular basis of SHP cytoplasmic and nuclear function.

## Results

### SHP Protein is Localized on the Mitochondrial Outer Membrane

**Figure 1:**
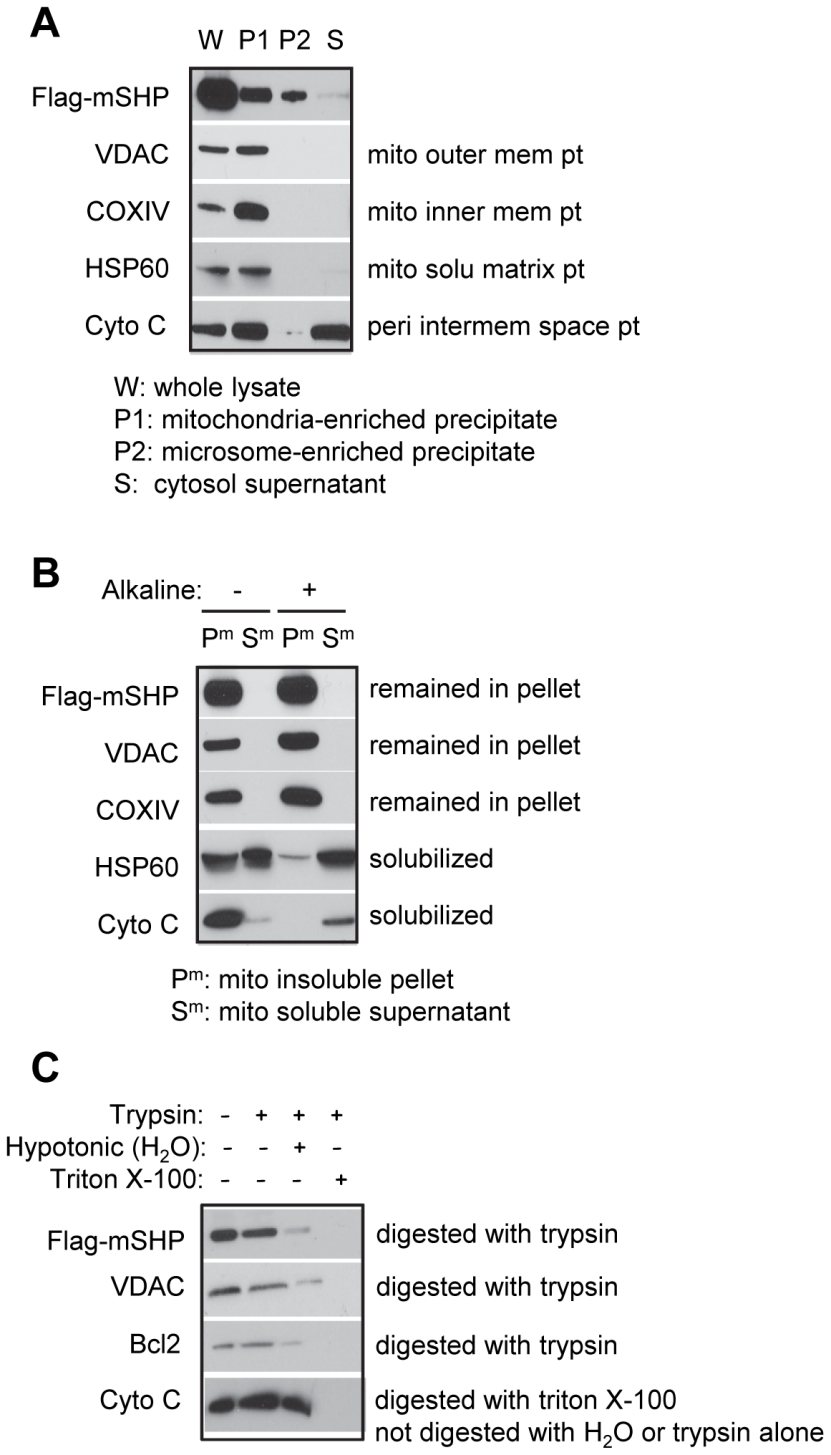
SHP protein is localized on the mitochondrial outer membrane. (A) Western blots to detect Flag-mSHP, VADC, COXIV, HSP60, and Cyto C proteins using specific antibodies for each protein, with the exception of SHP, which was detected with an anti-Flag antibody. Huh7 cells were transfected with Flag-mSHP (20 µg, 15 cm plate), and five plates were used to harvest protein. Differential centrifugation was used to isolate the mitochondria-enriched precipitate fraction (P1), microsome-enriched precipitate fraction (P2), and cytosolic supernatant fraction (S); the whole cell lysate (W) was used for comparison. (B) Alkaline digestion of mitochondrial membrane fraction (P^m^) and soluble fraction (S^m^) and Western blots to determine Flag-mSHP, VADC, COXIV, HSP60, and Cyto C proteins. (C) Trypsin protection assays and Western blots to determine Flag-mSHP, VADC, Bcl2, and Cyto C proteins in mitochondria. Abbreviations: mito, mitochondria; mem, membrane; solu, soluble; peri, peripheral; pt, protein.

Flag-mSHP plasmids were transfected into Huh7 cells, and different subcellular fractions, including whole cell lysate (W), mitochondria-enriched precipitate fraction (P1), microsome-enriched precipitate fraction (P2), and cytosolic supernatant fraction (S), were isolated by differential centrifugation. An anti-Flag antibody was used to determine SHP protein expression by Western blots. To judge the efficiency of sub-cellular fractionation of mitochondrial proteins, the fractions were subjected to immunoblots with antibodies against mitochondrial marker proteins. The mitochondrial marker proteins were highly enriched in the expected fractions, demonstrating efficient and clean separation of the outer membrane, intermembrane space, and inner membrane fractions. SHP was highly expressed in mitochondria enriched P1 (Fig. 1A), which co-localized with other known mitochondrial protein markers, such as VADC (mitochondria outer membrane protein), COXIV (mitochondria inner membrane protein), HSP60 (mitochondria soluble matrix protein), and cytochrome *c* (Cyto *C*, peripheral intermembrane space protein), confirming the mitochondrial localization of SHP. Interestingly, SHP also showed a robust enrichment in the P2, a microsomal fraction [29], [30], which was not seen with other mitochondrial proteins. A significant amount of Cyto *C* was detected in the cytosol as well, which was in agreement with our recent finding that overexpression of SHP induces Cyto *C* release [20].

We next isolated the mitochondria insoluble pellet (P^m^) and soluble supernatant (S^m^) fraction and treated them with alkali. In this experiment the integral mitochondrial membrane proteins VDAC (outer membrane) and COXIV (inner membrane) were retained in the membrane fraction (Fig. 1B). In contrast, the mitochondrial soluble matrix protein HSP60 was found in both P^m^ and S^m^ before alkaline treatment and was highly solubilized into S^m^ by alkali. As expected, Cyto *C*, a peripheral intermembrane space protein, was mostly observed in P^m^ but released upon alkaline extraction. The SHP protein, on the other hand, was present in P^m^ and was resistant to extraction by alkali and remained in the pellet along with VADC and COXIV, suggesting integration into the membrane lipid bilayer.

We further examined the association of SHP with the mitochondrial membrane by digesting purified mitochondria with trypsin. Similar to the mitochondrial outer membrane proteins VDAC and Bcl2, the SHP protein was digested by trypsin alone (Fig. 1C). In contrast, the soluble protein located in the mitochondrial intermembrane space, Cyto *C*, was sensitive to trypsin only in the presence of the detergent Triton X-100. Thus, SHP is associated with the outer membrane rather than being soluble in the intermembrane space. SHP contains numerous arginine (sixteen) and lysine (six) residues (*i.e.* trypsin cleavage sites) distributed throughout the polypeptide. We conclude, therefore, that the high sensitivity of SHP protein to trypsin arises because the mitochondrial distribution of SHP is largely restricted to the outer membrane with a domain of the protein exposed to the cytosol.

### Deletion of the SHP N-terminal Fragment Increases SHP Protein Expression in Mitochondria

Three programs (http://ihg.gsf.de/ihg/mitoprot.html; http://urgi.versailles.inra.fr/predotar/predotar.html; http://www.cbs.dtu.dk/services/TargetP/) were used to predict the mitochondrial targeting sequence (MTS) of SHP, and two programs (http://www.enzim.hu/hmmtop/html/submit.html; http://www.ch.embnet.org/software/TMPRED_form.html) were used to predict the transmembrane domain (TMD). However, no consensus was obtained.

**Figure 2:**
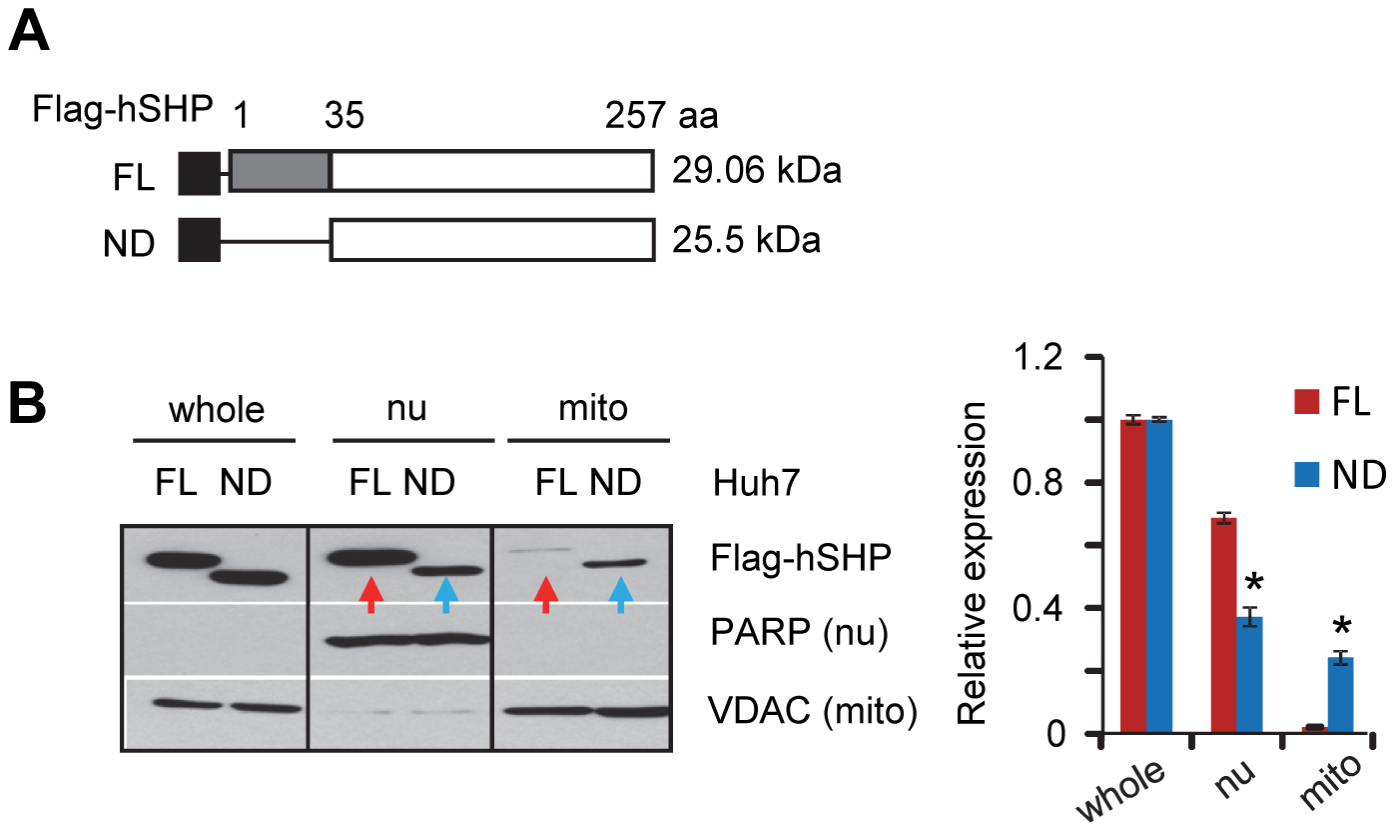
Deletion of the N-terminal fragment increases SHP protein levels in mitochondria. (A) Diagram showing the deletion mutation constructs of Flag-hSHP. FL, full length; ND, N-terminal deletion. (B) Left, Western blots to determine SHP protein expression in different cellular fractions in Huh7 cells. Right, band intensities were measured by densitometry, and the intensities relative to that of the whole cell lysate (set as 1) were plotted. Data are represented as mean ± SEM (*, *P*<0.01). Abbreviations: whole, whole cell lysate protein; nu, nuclear protein; mito, mitochondria protein. PARP is a marker for nuclear proteins; VDAC is expressed in mitochondria.

To experimentally identify the domain that is critical for SHP mitochondrial localization, we generated an N-terminal deleted Flag-hSHP construct and examined protein expression in the nucleus and mitochondria (Fig. 2A). The full length (FL) and N-terminal deleted (ND) Flag-hSHP proteins were expressed at similar levels (Fig. 2B, whole cell lysate). Interestingly, Flag-hSHP-ND protein exhibited lower expression in the nucleus (nu) but higher expression in the mitochondria (mito) relative to the Flag-hSHP-FL (Fig. 2B blue *vs.* red). The results suggest that SHP-FL protein with an intact N-terminus is expressed preferentially in the nucleus, whereas deletion of the N-terminus facilitates SHP mitochondrial expression.

### The SHP Interaction Domain is Primarily Responsible for Binding to Bcl2 in vitro

**Figure 3:**
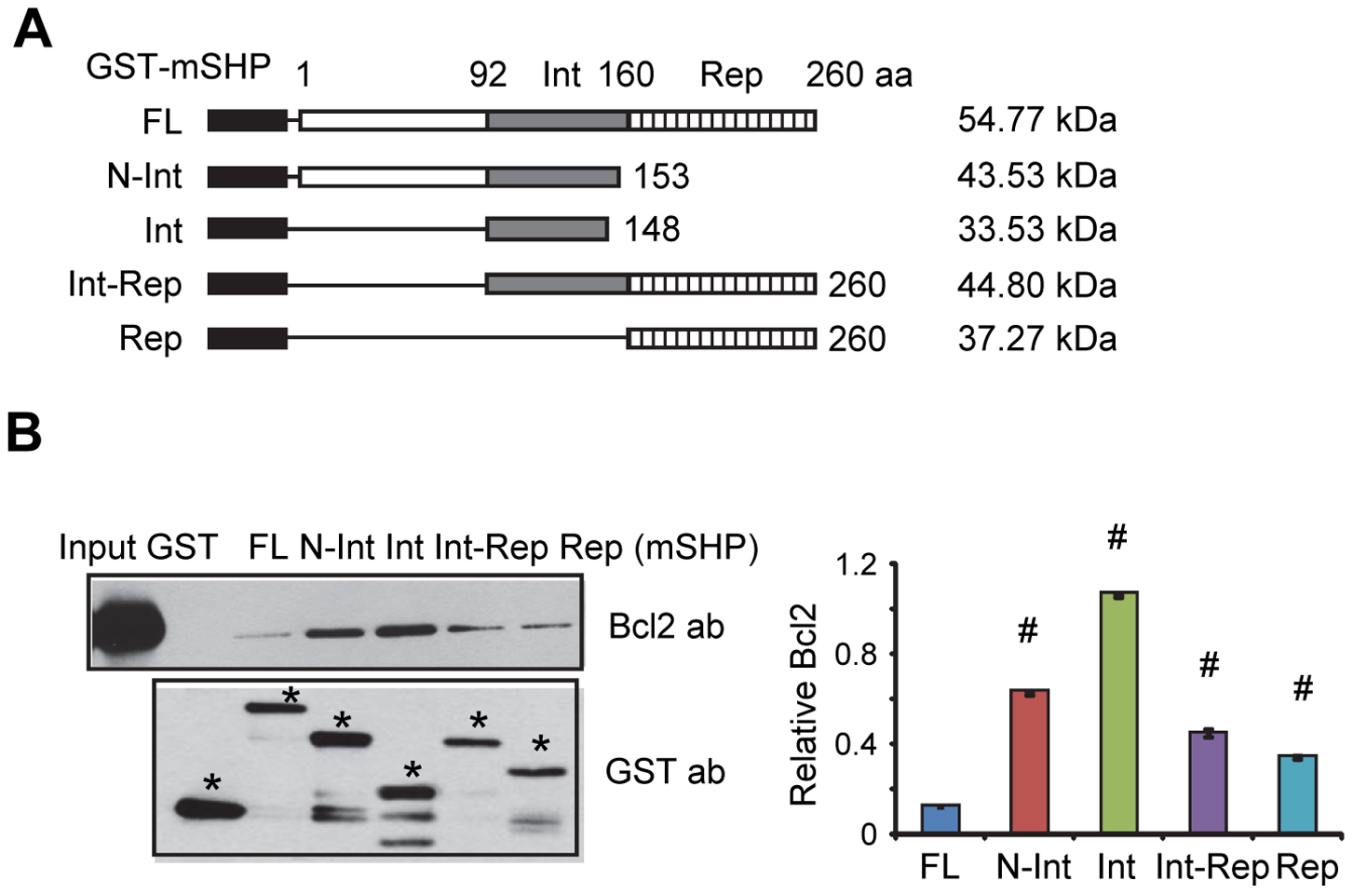
SHP interacts with Bcl2 primarily through its interaction domain *in vitro*. (A) Diagram showing GST-mSHP deletion constructs. (B) Left, GST pull-down assay to determine the *in vitro* interaction of SHP with Bcl2. GST or GST-mSHP full-length or deletion mutants used in the reactions were indicated by asterisks. The interaction of Bcl2 with GST-mSHP proteins was detected by Western blots using a Bcl2 antibody. Right, band intensities were measured by densitometry and normalized to each corresponding GST-mSHP fusion protein. Data are represented as mean ± SEM (#, *P*<0.01). Abbreviations: FL, full length; N, N-terminal domain; Int, interaction domain; Rep, repression domain.

Using Co-immunoprecipitation (Co-IP) and Western blots (WB) we recently showed that SHP and Bcl2 co-localized on the mitochondria [20]. However, the detailed interaction domains between SHP and Bcl2 have not been characterized. To analyze which domain of SHP directly interacts with Bcl2, we performed *in vitro* GST pull-down assays. Five GST-SHP constructs comprising full length (FL), N-terminal (N), interaction (Int), and repression (Rep) domains of SHP (Fig. 3A) were used to generate various GST-SHP fusion proteins and to determine their interaction with *in vitro* translated Bcl2.

This analysis revealed a direct SHP-Bcl2 protein-protein interaction (Fig. 3B). The strongest interaction was observed with the construct composed of the Int domain. The interaction of Bcl2 with N-Int was comparable with Int, suggesting that the presence of the N-terminal region did not strongly interfere with the interaction. The interaction of either FL with Bcl2, or Int-Rep with Bcl2, was significantly weaker, suggesting that the presence of the Rep domain may lessen the interaction between Int and Bcl2. Indeed, a weak band was observed with a construct containing only the Rep domain. Together, the results suggest that Bcl2 primarily interacts with SHP Int, but interacts weakly with SHP Rep; the latter likely functions to repress the interaction of Bcl2 with SHP Int.

### The Transmembrane Domain of Bcl2 is Mainly Responsible for the Interaction with SHP in vitro

**Figure 4:**
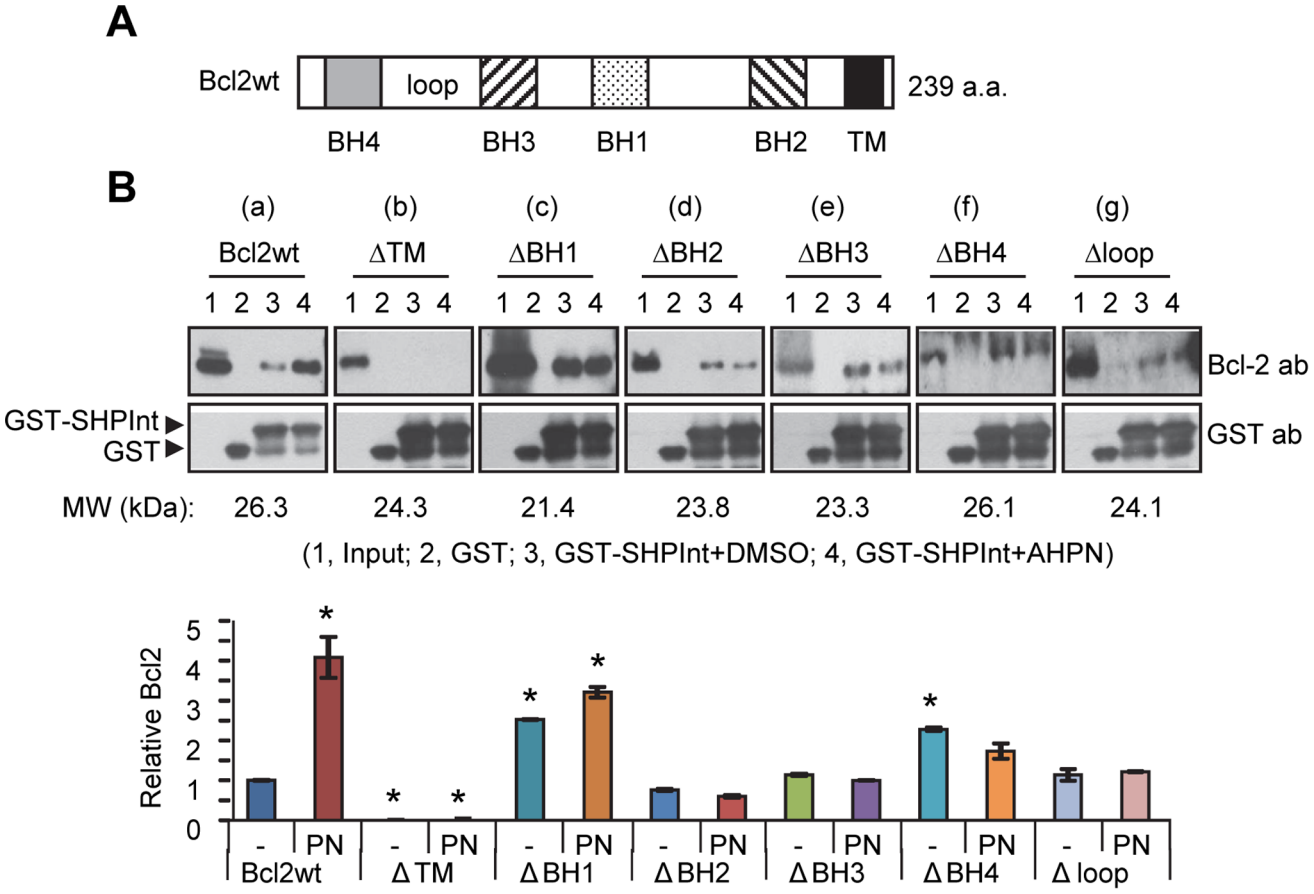
The transmembrane domain of Bcl2 is critical for its interaction with SHP. (A) Diagram showing the domain structure of Bcl2 protein. (B) Top, GST pull-down assay to determine the interaction domain of Bcl2 with SHP and the effect of AHPN on the *in vitro* interaction of Bcl2 with SHP. Bcl2wt, ΔTM, ΔBH1, ΔBH2, and ΔBH3 proteins were detected using an antibody against the N-terminus of Bcl2. ΔBH4 and Δloop plasmids contain a myc epitope, and these proteins were detected using an anti-Myc antibody. Bottom, Bcl2 band intensities were measured by densitometry, and the values were normalized by dividing by the GST-SHP Int intensities. The values for intensities relative to Bcl2 wt (set as 1) were plotted. Data are represented as mean ± SEM (*, *P*<0.01). Abbreviations: BH, BH domain; TM, transmembrane domain.

To address which domain of Bcl2 interacts with SHP, *in vitro* translated protein fragments of Bcl2 containing wild-type (wt) and various domain deletions (Fig. 4A) [31] and the GST-SHP Int were used in a GST pull-down assay. AHPN (retinoid 6-[3-(1-adamantyl)-4-hydroxyphenyl]-2-naphthalene carboxylic acid) was established as a SHP synthetic ligand [32] which also increased SHP mitochondrial translocation and enhanced SHP Co-IP with Bcl2 in the mitochondria [20]. Thus, we examined whether AHPN (PN) could affect a direct protein interaction between Bcl2 and SHP *in vitro*.

As expected, a direct physical interaction between Bcl2 wt and SHP proteins was observed, which was markedly enhanced by PN treatment (Fig. 4B-a, lane 4 *vs.* 3). Deletion of the transmembrane domain (ΔTM) of Bcl2 essentially abolished its association with SHP (Fig. 4B-b), indicating that the TM domain of Bcl2 is indispensable for the interaction. In contrast, the deletion constructs ΔBH2, ΔBH3, ΔBH4, and Δloop did not exhibit a diminished interaction with SHP (Fig. 4B-d-g), whereas the deletion in ΔBH1 strongly augmented the interaction of both proteins (Fig. 4B–c). Interestingly, the interaction of Bcl2 mutants with SHP protein was not altered by AHPN (Fig. 4B-c-g, lane 4 *vs.* 3), suggesting that AHPN is only effective with the intact Bcl2 wt protein.

### The N-terminal Domain of SHP is Critical for SHP Nuclear Translocation via HNF4α

We previously illustrated that in hepatoma Huh7 cells HNF4α facilitated SHP shuttling from the mitochondria to the nucleus [20]. However, it remained unclear whether such regulation occurs in other cell types and which domain of SHP is critical for nuclear translocation via HNF4α. The hSHP protein was expressed from a GFP-hSHP plasmid so that the GFP signal could be used as a marker to monitor SHP subcellular localization.

**Figure 5:**
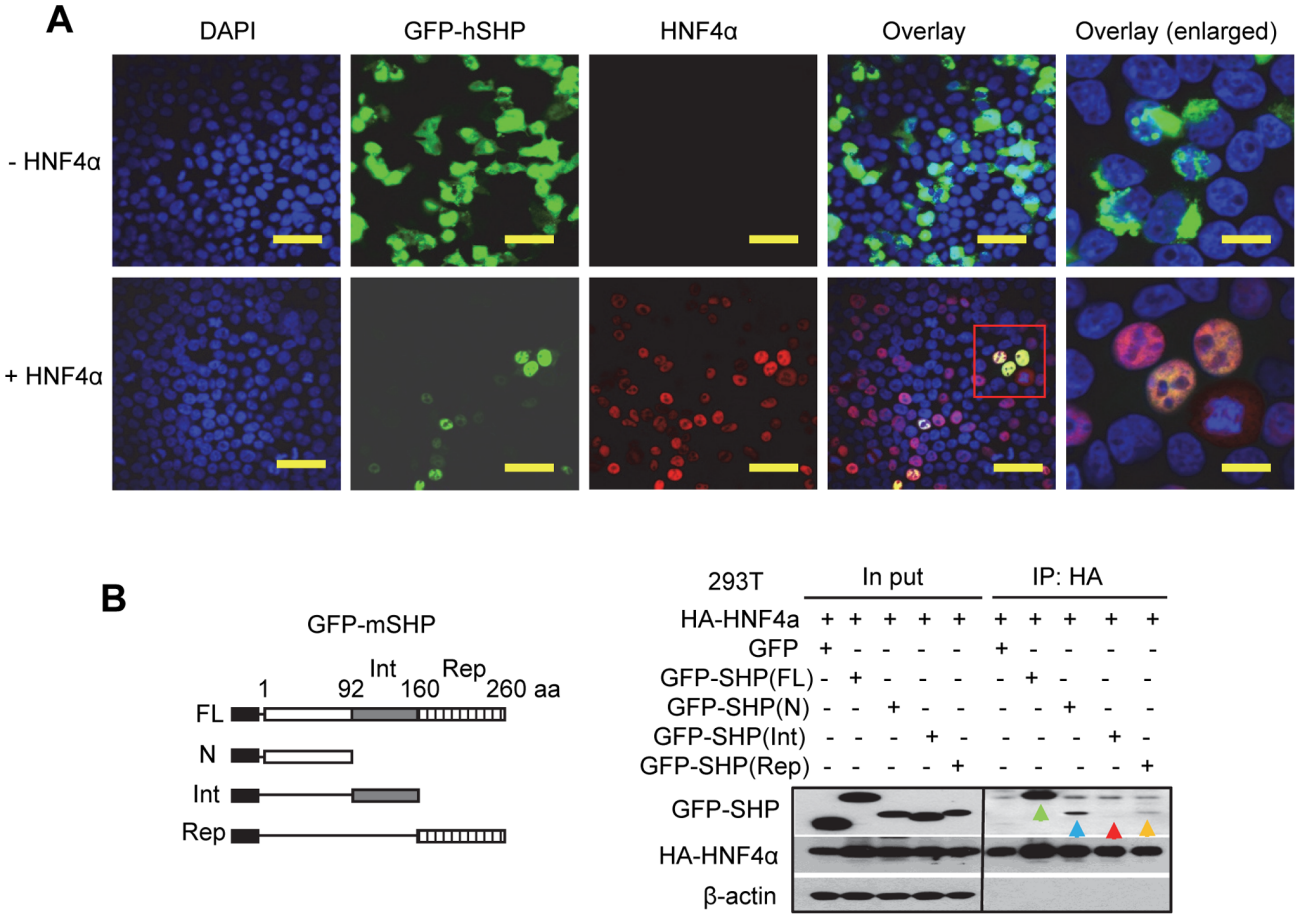
Ectopic expression of HNF4α induces an exclusive SHP nuclear translocation. (A) The subcellular localization of GFP-hSHP in 293T cells was observed under a fluorescence microscope, and HNF4α was visualized by immunofluorescence using a specific HNF4α antibody. Scale bars: 100 µm, 50 µm (insets). (B) The N-terminal domain of SHP is primarily responsible for the interaction with HNF4α protein. Left, Diagram showing the deletion mutation constructs of GFP-mShp. Right, 293T cells were transfected with GFP-mSHP deletion constructs (4 µg, 6 cm plate) along with HA-HNF4α (4 µg, 6 cm plate) expression vector. Anti-HA agarose was used to immuneprecipitate HNF4α, and the protein levels of various GFP-mSHP truncation mutants and HNF4α were detected by Western blots using anti-GFP or anti-HA antibodies, respectively. Abbreviations: FL, full length; N, N-terminal domain; Int, interaction domain; Rep, repression domain.

In the absence of exogenously expressed HNF4α in 293T cells, GFP-hSHP proteins were distributed in both cytosol and nucleus (Fig. 5A, top); the latter could be due in part to the endogenous HNF4α. Ectopic co-expression of HNF4α caused an exclusive SHP nuclear translocation (Fig. 5A, bottom). This is in good agreement with recent, independently derived results in Huh7 cells [20].

To further dissect out the domain of SHP that is important for its nuclear translocation, we generated GFP-mSHP deletion constructs containing the N-terminal, interaction (Int) or repression (Rep) domain, respectively (Fig. 5B, left). GFP-mSHP(N), GFP-mSHP(Int), and GFP-mSHP(Rep) proteins were expressed at ∼50% lower levels compared with GFP-mSHP(FL) (Fig. 5B, right). Co-IP and Western blots revealed that GFP-mSHP(FL) displayed the strongest association with HNF4α protein (green arrow), followed by GFP-mSHP(N) (navy arrow). GFP-mSHP(Rep) exhibited a weak interaction (yellow arrow). To our surprise, the protein interaction of GFP-mSHP(Int) with HNF4α was barely visible (red arrow); a stronger signal may likely be observed upon a longer exposure time. Overall, the results suggest that the entire SHP protein is important to maximize its interaction with HNF4α, and HNF4α primarily interacts with GFP-mSHP(N), but interacts weakly with GFP-mSHP(Rep).

**Figure 6:**
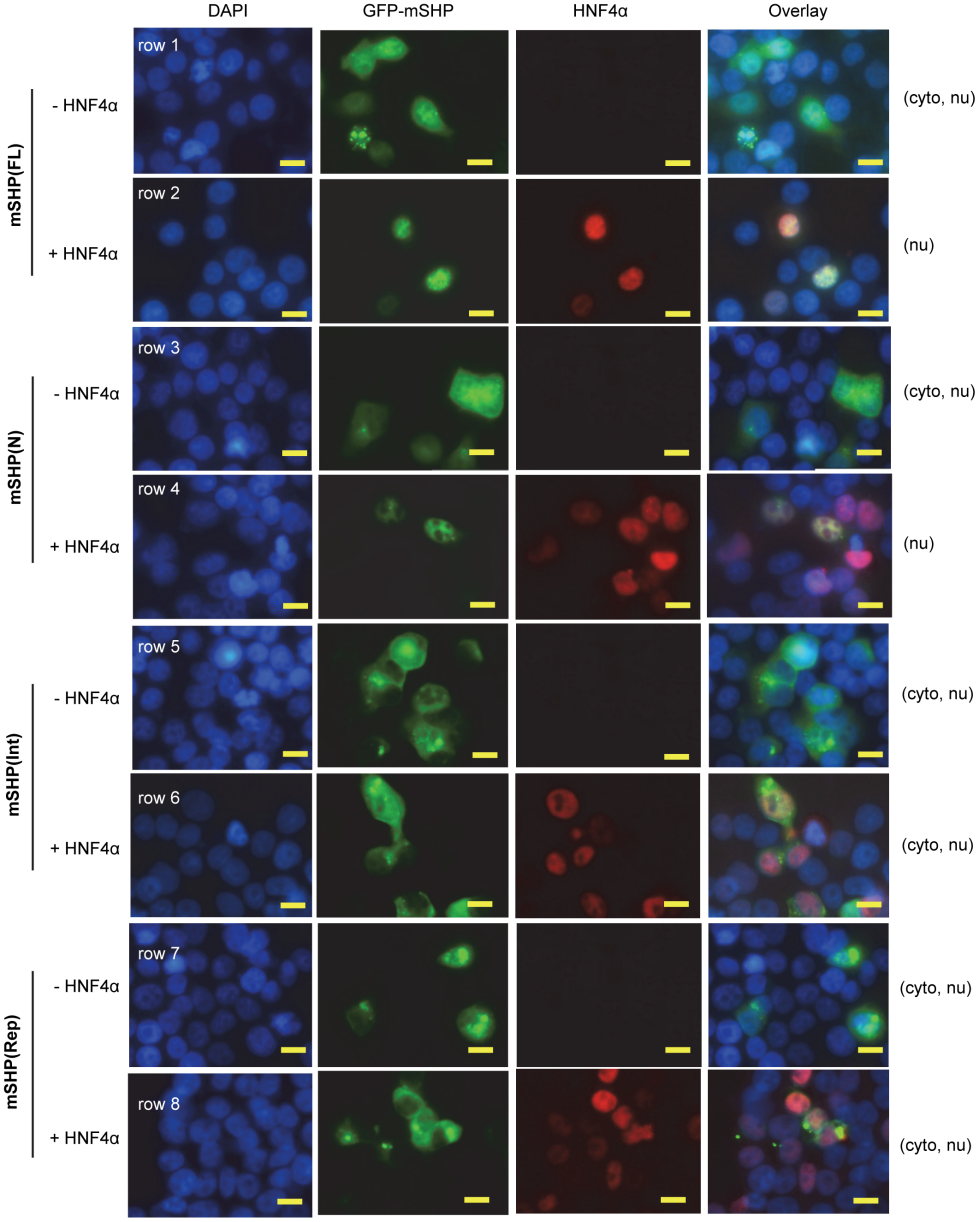
SHP nuclear translocation depends on its N-terminal domain. The subcellular localization of GFP-mSHP full length and deletion mutants in 293T cells was observed by fluorescence microscopy, and HNF4α was visualized by immunofluorescence using a specific HNF4α antibody. Scale bars: 50 µm. Abbreviations: FL, full length; N, N-terminal domain; Int, interaction domain; Rep, repression domain.

We next assessed nuclear translocation of GFP-mSHP(FL), GFP-mSHP(N), GFP-mSHP(Int), and GFP-mSHP(Rep) proteins in the absence or presence of exogenously expressed HNF4α. Similar to the GFP-hSHP protein (Fig. 5A), GFP-mSHP(FL) protein was distributed in both the cytoplasmic and nuclear compartments (Fig. 6, row 1), and co-expression of HNF4α induced an exclusive GFP-mSHP(FL) nuclear localization (row 2). GFP-mSHP(N) (row 3), GFP-mSHP(Int) (row 5), and GFP-mSHP(Rep) (row 7) proteins were also observed in both the cytosol and nucleus. Ectopic expression of HNF4α resulted in a complete GFP-mSHP(N) nuclear translocation (row 4), but had no significant effect on GFP-mSHP(Int) (row 6) or GFP-mSHP(Rep) protein cellular distribution (row 8). The results suggest that the N-terminal domain of SHP is critical for enhanced nuclear translocation through HNF4α.

## Discussion

Our previous study demonstrated that both the mouse and human SHP proteins are able to translocate to the mitochondria [20]. To gain more insight into the role of SHP in regulating mitochondrial function we determined the membrane localization of SHP. Despite the lack of a consensus mitochondrial targeting sequence, biochemical analyses provide solid evidence that SHP is concentrated in the outer membrane of mitochondria, although our results do not rule out the possibility that a small amount of SHP protein may also be located in the inner membrane.

SHP is known to interact with other NRs through its interaction domain [3], [4], which also appears to be the primary domain for the interaction of SHP with Bcl2. Interestingly, although Bcl2 works through the loop region to interact with another nuclear receptor, Nur77/TR3 [31], the TM domain of Bcl2 is vital for its interaction with SHP. Because the TM domain of Bcl2 has been reported to be responsible for its localization in the mitochondrial membrane [33], it is puzzling how this same domain can govern the interaction with SHP. An attractive hypothesis is that SHP and Bcl2 bind to each other in the cytosol prior to their translocation to the mitochondria. We are currently studying the role of Bcl2 in regulating SHP protein expression.

Of note, upon N-terminal deletion SHP protein expression in mitochondria is elevated and expression in the nucleus is decreased, suggesting that the N-terminus of SHP favors nuclear translocation. Previous studies showed that the two functional LXXLL-related motifs located in the N-terminal helix 1 (21–25 aa) and C-terminal helix 5 (118–123 aa) are important for SHP binding to a variety of nuclear receptors [3]. Indeed, the N-terminal third of SHP covering the front LXXLL-like motif shows stronger binding to HNF4α than other domains. Consistent with this observation and the results obtained in COS7 and Huh7 cells [20], [34], ectopic expression of HNF4α facilitates SHP protein nuclear translocation in 293T cells too, as long as the N-terminal region is intact.

An interesting observation is that in both Huh7 [20] and 293T (present study) but not COS7 [20], [34] cells, there is some nuclear localization of intact SHP even in the absence of HNF4α co-expression. This can be readily attributed to the endogenous expression of HNF4α in the first two cell lines. More intriguing is the finding in the present study that the partial nuclear localization of the SHP repression and interaction domains is not enhanced by exogenous HNF4α. This may reflect a role for other NRs that interact with SHP to promote its nuclear translocation. Overall, in addition to clarifying some of the key elements of SHP subcellular localization, this study provides a number of exciting observations for future exploration of the role of SHP in mitochondrial function and metabolism.

## Materials and Methods

### Plasmids and Reagents

The cDNAs of the wild-type mouse Shp (Gene ID: 23957) and human SHP (Gene ID: 8431) were cloned in the expression plasmid pcDNA3-GFP and pCMV-Flag, respectively, as we described previously [35]. The Flag-hSHP FL (full length) and Flag-hSHP ND (N-terminal deletion) plasmids were generated using the following primers:

hSHP FL+: CGGAATTCATGAGCACCAGCCA.

hSHP FL-: CTAGTCTAGATCACCTGAGCAAAAG.

hSHP ND+: CGGAATTCCGTAGCCGCTGCCTAT.

hSHP ND-: CTAGTCTAGATCACCTGAGCAAAAG.

The GFP-mSHP FL (full length), GFP-mSHP N (N-terminal domain), GFP-mSHP Int (interaction domain), and GFP-mSHP Rep (repression domain) plasmids were generated using the following primers:

mSHP FL +: CGGAATTCCGAATGAGCTCCGGCCAGT.

mSHP FL −:GCTCTAGAGCTCACCTCAGCAAAAGC.

mSHP N+: CGGAATTCCGAATGAGCTCCGGCCAGT.

mSHP N −: GCTCTAGAGCTCATAGCAGCCGCCGCTGAT.

mSHP Int +:GGAATTCCGAGAGTGCTGCTGGGGCCCTCTCTT.

mSHP Int −:GCTCTAGAGCTCAGAAAGACTCCAGGC.

mSHP Rep +:CGGAATTCCGATGGAGCCTTGAGCTG.

mSHP Rep −:GCTCTAGAGCTCACCTCAGCAAAAGC.

The wild-type HNF4α plasmid was obtained from Dr. Francis Sladek (UCR). The GST-SHP constructs were obtained from Dr. Kim Kemper (UIUC). Various Bcl2 constructs were generously provided by Dr. Xiaokun Zhang at the Sanford Burnham Medical Research Institute [31]. The VDAC antibody (#4661), COX IV antibody (#4850), Hsp60 antibody (#4870), Cyto c antibody (#4280), PARP antibody (#9532), Bcl2 antibody (#2870), myc antibody (#2278), GFP antibody (#2955), GST antibody(#2625), and HNF4α antibody (#3113) were purchased from Cell Signaling. Flag antibody (#F1804) and β-actin antibody (#A1978) were purchased from Sigma. Bcl2 antibody (#ab7973) was purchased from Abcam.

### Cell Culture and Transfection

The human hepatoma cell line Huh7 (Japan Health Science Research Resources Bank, #JCRB0403) and human embryonic kidney cell line HEK293 (ATCC, #CRC-1573) were maintained in Dulbecco’s modified Eagle’s medium (DMEM) with 10% fetal bovine serum. Transfection was performed by Lipofectamine 2000 (Invitrogen) according to the manufacturer’s instructions.

### Biochemical Analysis of SHP Localization in the Mitochondria

Subcellular fractionation: Fractionation of Huh7 cells was performed according to the methods described by Kuroda [29] and Horie [30]. Briefly, cells in 15-cm dishes were transfected with 20 µg Flag-mSHP plasmid, harvested at 48 h post transfection, and disrupted in isotonic buffer (phosphate buffered saline containing 0.2 M mannitol, 0.07 M sucrose, and 1 mM EDTA) containing protease inhibitors (#78410, Thermo Fisher Scientific Inc., Rockford, IL, USA), followed by centrifugation at 800 g at 4°C for 10 min to obtain a post-nuclear supernatant (PNS). The PNS was centrifuged at 17,000 g at 4°C for 10 min to obtain the mitochondria-enriched precipitate fraction (P1). The supernatant was centrifuged at 170,000 g at 4°C for 30 min to separate the microsome-enriched precipitate (P2) and supernatant fractions (S). The subcellular fractions were subjected to SDS-PAGE and then analyzed by Western blots.

Alkaline treatment of mitochondria: Mitochondrial protein (100 µg) prepared from Huh7 cells expressing the Flag-mSHP proteins was treated with 100 mM Na_2_CO_3_ in 10 times the volume of mitochondrial suspension for 1 hr on ice. The reaction mixtures were centrifuged at 170,000 g at 4°C for 30 min to separate the precipitate (P^m^) and supernatant (S^m^) fractions. The fractions were subjected to SDS-PAGE followed by Western blots.

Trypsin protection assay: the mitochondria were treated with either H_2_O or 2% triton X-100 in 10 times the volume of mitochondrial suspension on ice for 1 hr, and then treated with 50 µg/ml trypsin on ice for 1 hr. The reaction mixtures were separated by SDS-PAGE and analyzed by Western blots.

### Glutathione S-transferase (GST) Pull-down Assays

The MagneGST™ Pull-Down System (#V8872, Promega, Madison, WI) was used in this experiment. Detailed information can be found in our previous publication [35]. In brief, *in vitro* synthesis of various Bcl2 proteins was performed using the TNT-T7 quick coupled transcription/translation system utilizing the T7 promoter (#LI170, Promega, Madison, WI) according to the manufacturer’s instructions. GST, GST-SHP, or GST-SHP deletion mutants were expressed in *Escherichia coli* BL21/DE3/RIL. For pull-down experiments, bacterially expressed GST fusion proteins were bound to glutathione-containing MagneGST™ particles, and the binding of the *in vitro* translated Bcl2 protein with GST fusion protein was conducted using the MagneGST™ pull-down system as described in the manufacturer’s manual. The bound proteins were separated by SDS-PAGE, followed by Western blotting with the indicated antibodies according to the standard procedures.

### Immunofluorescence Assay

Cells were cultured overnight on coverslips and were transfected with various plasmids as required. Cells were washed twice with PBS, fixed in 3.7% paraformaldehyde for 20 min at room temperature (RT), and then permeabilized in chilled methanol for 3 min at RT. After washing with PBS, the cells were incubated with blocking solution containing 5% bovine serum albumin (BSA) in PBS for 1 hr at RT. To identify HNF4α protein, cells were incubated first with anti-HNF4α antibody diluted in 5% BSA–PBS overnight at 4°C, washed with PBS, then incubated with the corresponding Alexa Fluor 592 goat anti-rabbit IgG (1∶200; Molecular Probes) for 30 min at RT. Cells were then washed in PBS, and coverslips were mounted onto slides with Prolong Gold antifade reagent (p36930; Molecular Probes Inc., Eugene, OR). Immunofluorescence images were captured on an Olympus fluorescent microscope (Olympus BX51; Olympus America Inc., Melville, NY).

### Co-immunoprecipitation (Co-IP)

HEK293T cells were transfected with the indicated plasmids, lysed in 500 µl lysis buffer, and immunoprecipitated with monoclonal anti-HA agrose (Sigma) overnight at 4°C. The agrose was washed four times with the lysis buffer. The bound proteins were separated by SDS-PAGE, followed by Western blotting with the indicated antibodies according to the standard procedures.

### Statistical analysis

Data are expressed as mean ± SEM. Statistical analyses were carried out using Student’s unpaired t test; p<0.05 was considered statistically significant.
